# Supporting the integration of first-year undergraduate widening participation sport students into university: the role of online programme induction

**DOI:** 10.1007/s43545-022-00574-7

**Published:** 2022-12-06

**Authors:** Rick Hayman, Michael Wood, Karl Wharton, Lynette Shotton

**Affiliations:** 1 Department of Sport, Exercise and Rehabilitation, Northumbria University, Newcastle, UK; 2 Department of Social Work, Education and Community Wellbeing, Northumbria University, Newcastle, UK

**Keywords:** Higher education, Online induction, Student engagement, Transition, Widening participation

## Abstract

Programme induction activities are a central feature for supporting successful student entry into university, playing an important role in ensuring they quickly settle, feel included, are motivated to learn and able to form new friendships and networks with peers and staff. Research shows how entering university can be complex and challenging for all students regardless of background and experience. This is particularly the case for widening participation students, who often encounter excessive social exclusion and financial pressures. By using Student Involvement Theory (Astin in J Coll Stud Pers 25:297–308, 1984) as a guiding theoretical framework and peer mentors as interviewees, the primary aim of this study was to explore the effectiveness of an exclusively online programme induction in supporting the integration of newly arrived first-year widening participation sports students into a post-92 British university. The key study finding was that online induction was more successful in gaining academic than social engagement. Participants devoted time and effort into their studies but had limited social involvement with other students, both from their programme and the wider university community. Practical implications for developing future online induction programme schedules to better support the transition of diverse student populations into university are presented, as are future research avenues and limitations.

## Introduction

Higher education (HE) is internationally recognised for its positive impact in shaping life satisfaction, personal development and prosperity (O`Shea et al. 2018). Increasing the diversity of learners who enter HE has been central to the British government’s political drive for addressing inequality of HE access for under-represented student groups (Conell-Smith and Hubble 2018; Moore et al. 2013; Thompson 2019). This widening participation (WP) agenda has achieved relative success over recent decades, evidenced by the steady growth in proportions of the British population from non-traditional and socio-economically disadvantaged backgrounds gaining an undergraduate degree (Kneta and McCartney 2018). This includes those classified as, first generation, part-time, disabled, mature and individuals entering from low-income households or deprived neighbourhoods (Younger et al. 2018).

The British HE sector remains committed to further increasing student diversity and for many institutions remains a key part of their student recruitment drives (Harrison and Hatt 2012; Jones and Lau 2010). The WP agenda has challenged the concept of what it now means to be a university student, moving away from traditional views where students were often constructed as young, white males from upper or middle-class backgrounds (Chung et al. 2017). In England, for example, application rates for 18 year olds living in areas with historically low HE participation rates increased to the highest recorded levels in 2018 (Universities UK 2018), whilst over half of all school leavers in Scotland now enter HE (Tett et al. 2017).

### Transitioning into university

Studies across a range of national and international contexts have made it clear how the transition for students from school or college into HE can be a complex and non-linear process (Gravett and Winstone 2021; Hayward et al. 2006; Richardson and Tate 2013; Taylor and Harris-Evans 2018) which some groups manage better than others (Coertjens et al. 2017; Gale and Parker 2012; Parker et al. 2017). From a WP student perspective, this transition period can be even more difficult because of the many socio-cultural and economic differences they encounter which may lead to unique issues and challenges (Parker et al. 2017; Read et al. 2003; Young et al. 2020). Research shows how WP students have regularly encountered very different transitions into HE, and experiences within it, than their peers from higher economic status backgrounds (Pittman and Richmond 2007; Iyer et al. 2008; Reay et al. 2010), often having to operate under extreme financial pressures (Crozier et al. 2008) and more likely to encounter discrimination, disruption and a degree of social exclusion (Reay 2018).

It is also well established, but especially for WP students, that early withdrawal rates are higher during the first 12 months of enrolment than at any other stage of their studies (Berger et al. 2012; Kahu and Nelson 2018; Tinto 2010) and that this is strongly associated with unsuccessfully integrating and overcoming various academic and non-academic barriers (Devlin 2013; Parker et al. 2017; Smith and Naylor 2001). This is especially the case for those who enter from families who have not previously attended university and may be lesser equipped to master the dominant cultural codes and practices in HE, which ‘traditional’ students may have readily obtained through their prior social, cultural and educational experience (Chung et al. 2017). These findings can be explained by Bourdieu’s notable works in the sociology of education and his concerns with social class and social reproduction (Bourdieu and Passeron 1990). Bourdieu suggests education systems are based on the assumption that learners are in possession of cultural capital, which for WP students from lower social class backgrounds—or families where there has been no previous experience of university education—may be lacking. This often means that students from such backgrounds are disadvantaged (Sullivan 2001), believing that they lack the behaviours, skills or ways of interacting necessary for university success (Ivemark and Ambrose 2021).

Successful transition into HE is reflective of newly arrived students feeling they have settled promptly and happily into their course and wider university community, made new friends and networks, have developed a sense of belonging and identity with peers and academic staff and are motivated to learn (Farhat et al. 2017; Thompson et al. 2021). The seminal student engagement work by Tinto (1987) found that students new to university were likely to leave previous associations or activities behind and seek to forge new friendships and social relations to become part of communities associated with their new environment. These may include groups associated with campus life, halls of residence, societies, sport and/or academic learning. But an emerging evidence base shows how regardless of background and experience, students stepping into HE are more likely to disengage, underachieve and ultimately discontinue with their studies if they feel isolated and unsupported and when expectations are not fully met (Bradley 2017; Jensen and Jetten 2015; Turner et al. 2017; Young et al. 2020).

Such problems are exacerbated with the diversity of students now attending university, of which many no longer leave home, but instead commute onto campus daily. Berger (1997) found that friendships formed in halls of residences were important sources for developing a sense of community, with those not living in student accommodation more likely to feel marginalised. Wilcox et al. (2005) showed the importance of regular social support from peers and staff for coping with feelings of isolation, unhappiness and loneliness. Part-time, mature and working-class students also experience difficulties when entering into HE and have higher non-completion rates compared to those from traditional backgrounds (Rubin 2012; Tinto 2010). This concurs with Stevenson (2012) who found black and ethnic minority students’ felt more could be done to support their social integration into university, especially for those who did not drink alcohol.

Thomas (2012) found difficulties in socially integrating, including homesickness and inability to make new friends, as influencing factors for university withdrawal. Thompson et al. (2021) explored the experiences of newly arrived students at British universities, finding they struggled adapting to living self-reliantly, were unprepared for independent study and that new social networks were a key source of support but establishing these initial connections was challenging. Parker et al. (2017) explored challenges faced by students from diverse ethnicities when they transitioned into HE, finding they must be made to feel welcomed and supported to settle, integrate and gain confidence if they are to achieve successful outcomes.

Literature further demonstrates how first-year undergraduate entrants generally experience school and further education learning where study was more structured and tutor-led, with fewer teaching staff involved and where groups were small enough for everybody to know one another (Abrahams et al. 2016; Allin et al. 2017; Belfield et al. 2017). These students are more likely to have insufficient understanding of what HE level learning entails (Gamache 2002; Lowe and Cook 2003), lack appropriate academic study skills (Trotter and Roberts 2006), feel underprepared for the assessment methods they are likely to face (Allin et al. 2017; Fahrat et al. 2017) and are unaware that independent learning is a requirement for success in HE (Entwistle 2005; Hockings et al. 2018).

### Transitioning of sports students into university

ALLIN et al. (2017) undertook one of the few studies to have examined sports students encounters of entering HE, finding many arrived with a mixture of social and academic concerns about managing workload, completing assessments to the required academic standard, being away from home, missing family and pets, poor sense of preparedness and being independent. Hayman et al. (2020) undertook focus groups with 32 undergraduate sports students from WP backgrounds, finding student-centred personal tutoring played an important role in supporting them gain confidence and a sense of belonging to the institution. The motivations, perceived challenges and concerns of 334 first-year sport students at a British post-92 institution were explored by Hayman et al. (2021), who found participants lacking in confidence to integrate socially and academically into university. These studies illustrate how many newly arriving sports students now enter HE with uncertainties, fears and concerns about transition, workload, independence and responsibility. This is particularly the case for those entering from WP backgrounds who may require greater levels of guidance and support as they move into HE settings and learn to acclimatise with student life.

### Role of university induction

An inviting, well-designed and student-centred induction is crucial to the first impressions and individual success of every learner entering HE. It sets the tone and foundation for a high-quality student experience and can make the difference between retaining or losing students and their future progression and achievement (Edward 2003; Turner et al. 2017). A high-quality induction should be inclusive and accessible to all, introducing learners to their programmes of study and explaining principles, rules, regulations and other important academic and non-academic information that students will need to know so they feel well-prepared, motivated to learn and confident to form new friendship groups and relationships with academic staff (Turner et al. 2017). The findings of Edward and Middleton (2002) and Gaskin and Hall (2002) demonstrate how operating extended programme level induction periods increased readiness for study and social integration in engineering and geography students. Richardson and Tate (2013) argued in favour for extending the duration of first-year university induction to over several days and incorporating peer mentoring opportunities to help with developing social communities and preparedness for university learning.

### Research context

This study was conducted at an English post-92 university (hereafter referred to using the pseudonym DRM). The origins of DRM are rooted in the need to provide practical and vocational education, and this remains a core feature of its modern-day provision. DRM is renowned for teaching excellence and ensuring fair access and reducing educational inequality are key strategic outcomes underlined in both its 2020–2021 to 2024–2025 Access and Participation Plan and Corporate Strategy. When compared across the British HE sector, DRM has traditionally recruited higher student numbers from under-represented backgrounds, although this figure has fallen slightly since 2015. DRM has previously encountered retention issues with first-year sport student cohorts, especially those characterised as WP. Between 2015 and 2019, newly arrived sports students to DRM undertook an intensive 2-day face-to-face induction experience which placed heavy emphasis on supporting social and academic integration into university. This was achieved through students undertaking several team building, practical sport activities and social events, including indoor climbing, multi-skill games, ten-pin bowling and quizzes in small peer groups (15–20) and with course staff.

The primary aim was on having fun, developing social bonds, creating community, enhancing belonging and connectedness, setting standards and expectations and involving academic personnel to help develop preparedness, good habits and confidence for university life. Each academic year, the intake was approximately 200 students. The majority typically enter with vocational or level 3 foundation degree backgrounds, although some enter with a combination of BTEC^1^/A level and a small minority with A levels only. A significant proportion of new sports students now enter from backgrounds not typically considered traditional, including those who live at home and commute daily into university, enter with vocational qualifications, are from low-income families, are the first in their family to enter HE or who come from neighbourhoods where HE is not a common destination (Crosling et al. 2008; Turner et al. 2017).

The impact of the global Covid-19 pandemic challenged the appropriateness and long-term sustainability of the operating models, systems and practices within HE institutions, stimulating significant changes in the ways they supported the entry of newly arrived student cohorts. This was especially the case for the design and delivery of the 2020–2021 programme induction, which new intakes of DRM sport students experienced in late September 2020. Fundamentally, 2020–2021 programme inductions were restricted to a skeleton online programme which provided a range of course specific (e.g. overview of module content and navigation of Blackboard Ultra^2^), university generic (e.g. introductions from senior university management personnel) and pastoral information (overview of personal tutor role and health and well-being services and support). Timetabled over a 4-h period, students were expected to attend multiple online university and programme specific sessions. These were predominantly delivered using Blackboard Collaborate^3^ and Microsoft Teams by senior management colleagues and programme leaders, with occasional contributions from library, student union, academic registry, careers service, student support teams and alumni colleagues. Towards the later stages of induction week, students were required to formally meet online with their personal tutor, for which attendance was less than half.

### Theoretical framework

ASTIN (1984) proposed a student engagement theory based on student ‘involvement’, which he defined as ‘the amount of physical and psychological energy that students devote to the academic experience’ (Astin 1984, p. 297). The theory proposes how the more that students feel academically and socially involved in their studies, then the better their overall learning experience will be and the less likely they are to drop out or fail. Thus, within the context of Student Involvement Theory, a highly involved student is one who devotes considerable time and effort to studying, participates actively in a range of student organisations, clubs and societies and interacts frequently within and outside of their studies with other students and academic staff. Alternatively, a typically uninvolved student will display early signs of neglecting their studies, only rarely attend formal timetabled teaching sessions, withhold from participating in extra-curricular and social activities and have infrequent contact with fellow students and academic staff, both within and outside of class (Astin 1999).

Research supports Astin’s theory, with educators having used it to design educational practices to promote student learning and development (e.g. Hunt 2003; Morgan 2001). Pascarella and Terenzini (1991) and Rubin et al. (2002) both found students who were regularly involved in extra-curriculum activities had higher educational aspirations, enhanced self-confidence, and increased interpersonal, communication, teamwork and leadership skills than those peers who did not. Foreman and Retallick (2013) found the number of university clubs which students attended with positively associated with higher leadership scores. Research has also shown how WP university students, including those with commuter and employment commitments, can encounter lower levels of campus involvement than those who live in university accommodation and do not work (Alfano and Eduljee 2013; Newbold et al. 2011).

### Study rationale and aim

Whilst formal induction into HE is standard sector practice, there is no mandate to how this is conducted and despite its relative importance, students’ induction experiences and the resulting impact it has on supporting their transition into university life remains understudied. This is particularly the case for British-based sports students, which is surprising considering the large and diverse cohorts annually recruited to sports programmes from WP backgrounds. Using Student Involvement Theory (Astin 1984) as a guiding theoretical framework, the primary aim of this study was to explore the effectiveness of an exclusively online programme induction approach in supporting the integration of newly arrived first-year WP sports students at DRM. Findings will highlight if there is a future need for hybrid approaches to university programme induction which use multiple delivery approaches and capitalise on innovation in delivery to expand access to WP students (e.g. learners with disabilities or caring responsibilities who may find it difficult to attend face-to-face campus activities).

## Methodology

### Design

The study design provided opportunity for students to be engaged as partners and work alongside the research team at all stages. In total, 4 female second-year DRM undergraduate sport students were recruited and trained as peer mentors to conduct online semi-structured interviews. An online training session led by the first author took place in late November 2020 to support the peer mentors in carrying out the interview process and in defining their roles and boundaries within the study.

### Participants

During late October 2020, all newly arrived first-year undergraduate sports students were invited to participate in the study. Once institutional ethical clearance was granted, a recruitment email briefly outlining the study aims, objectives, inclusion criteria and procedures to follow, along with participant information sheet and consent form were communicated via the online Blackboard Ultra portal. This specifically explained how the research team were wishing to recruit and then interview participants via Microsoft teams, who were categorised in one or more of the following WP student criteria: (1) first generation, (2) commuter, (3) BTEC entry qualification, (4) Black, Asian Minority Ethnicity (BAME), (5) Mature (21 years or older).

A self-selecting sampling approach was used, resulting in 9 (male = 6; female = 3; mean age = 19.8) eligible full-time first-year undergraduate sport students agreeing to become participants (Table 1).

**Table 1:** Participant information

Participant	Background
1	First generation; BAME
2	First Generation; Commuter; BTEC entry
3	Mature, First Generation
4	First generation; BAME
5	First Generation; Commuter
6	Commuter, BTEC entry
7	First Generation, BTEC entry
8	First Generation, Commuter, BTEC entry
9	Mature; Commuter

The majority (78%) were the first from their immediate family to attend university. In total, 50% had prior completed a BTEC sport qualification either at college or school sixth form and 56% resided at home during the pandemic. All consenting participants were assigned numerical pseudonyms to protect anonymity and were free to withdraw from the study at any time.

### Procedure

Each interview was completed using Microsoft Teams at a convenient time for both interviewee and interviewer during December 2020 and lasted between 22 and 36 min. The interview schedule was pilot tested on two first-year sports students who both experienced the online programme induction at DRM but did not want their data to be included in the project. This confirmed an approximate completion time of 30 min, with all wording and terminology considered appropriate and understandable for first-year students. A copy of the finalised interview guide is available on request from the first author.

Each participant was offered the option to be interviewed by either a member of the research team, each with experience of undertaking qualitative educational research, or by a trained second-year peer mentor. In every case, interviews took place with a peer mentor. Byrne et al. (2015) suggest the use of peer interviewing tends to generate more honest and insightful discussions on sensitive topics. Consequently, it was felt that the second-year peer mentors involvement enabled first-year students to talk more freely and openly about their general online induction and transitional experiences at DRM. Informal conversations with peer mentors found they enjoyed the experience and that it supported the development of their own research skills and understanding of HE transitional barriers and challenges.

When undertaking qualitative interviews, it is important for the interviewer to quickly build trust and rapport with consenting interviewees, so they feel reassured, comfortable and relaxed to freely discuss topics they feel appropriate. The peer mentors were trained to undertake the role of ‘active listener’ during each interview so they could assist participants in telling their unique stories in their own particular manner. Using open-ended questioning, the first stage of each interview focussed on unpacking participant’s initial experiences of transitioning into DRM, with specific emphasis on the role played by their online programme induction. In the second stage, questions specifically explored their experiences of socially integrating into DRM without having encountered any traditional face-to-face teaching provision.

To elicit greater richness and meaning to responses, questions when necessary were supplemented by probes (Smith and Osborn 2003), enabling the direction of interviews to be guided by participants, rather than dictated by the schedule, and made it possible to follow-up any additional information discussed (Smith and Osborn 2003). Example interview questions included ‘were your expectations of an online programme induction realised’, ‘discuss your online program induction experience’, ‘what have been the most helpful activities and/or individuals in helping you to settle at DRM’ and ‘how would you rate your overall DRM online programme induction experience’. This flexible approach of questioning ensured participant centeredness, making it possible to follow up conversations where appropriate (Kornbluh 2015; Lincoln and Gubba 1985). Every attempt was made to understand the unique experiences and accounts of each participant rather than following a standardised list of questions.

### Analysis

Each interview was recorded, transcribed verbatim and subjected to the thematic analysis guidelines published by Braun and Clarke (2006). Every transcript was read multiple times by the first and fourth authors, with notes reflecting theme statements and their meanings placed within margins. The next stage involved the same authors independently annotating each interview transcript with their personalised interpretations of the data. Thematic coding employed an inductive approach to allow for lower order themes to be derived. There were some slight discrepancies between the two separate coding results, but all were discussed and swiftly resolved. Primary associations and connections based on similarities and patterns between derived themes were made, resulting in the generation of three main themes. Once finalised, direct quotes representing each theme were selected. The final analysis stage involved developing written accounts from identified themes which were reviewed and redrafted several times.

## Results

The findings are presented under three key themes which reflect the experiences and views of the study participants. Participant numbers are presented in parentheses (e.g. P1, reflects participant 1).

### Theme 1: Online induction provision as a platform for supporting academic integration

A primary aim of the online induction offer was to develop relationships between students, academics and support staff and produce a developing sense of academic community. All participants were still present at DRM at time of interview, expressing how their online programme induction played a key role in supporting their academic integration into the institution. Several discussed how it helped to grow their understanding of what university level learning entailed and made them feel better prepared for the teaching, learning and assessment approaches they were likely to encounter. The comments outlined below illustrate this way of thinking further:

To be honest, starting university has been better than I expected it to be considering we went into this year with Covid. I was expecting it to be a lot worse. I am doing alright academically, and I like my course. Induction was really good. (P3)

So far, I have found it quite good. At the start, I found it a bit challenging to try and settle in, because with it being online and the whole lockdown thing. But I feel after about a couple of weeks in, I got used to it and then got into the flow of the academic work (P5).

All participants actively engaged in all aspects of their online programme induction. Most described the overview of academic features on their course, as well as the computer systems involved (e.g. Blackboard Ultra) to have helped in supporting them to better understand the academic requirements of the programme. The emphasis and support placed by academic staff into encouraging new arrivals to learn independently and collaboratively with fellow peers during their university studies were well received. Providing information about the course, modules and academic staff, role of the personal tutor, an overview of the student portal, guidance on how to access and use email, electronic library and skill support mechanisms were also mentioned as being particularly useful. Participant 3 illustrated this by saying:

The induction helped in terms of each staff member creating a specific video about themselves and that was nice to see

Participants liked how online induction provided academic guidance and support on managing university workloads, developing effective habits of full-time study, balancing studies with work, caring and extra-curricular commitments, keeping organised and up to date with assessments, addressing required academic standards and in stimulating excitement about their subject area. Whilst adding to the escalating challenges of adjusting to university life upon entry from school or college, the constraints of the global pandemic meant participants were quickly required to become distance learners, to work independently and to be self-motivated. Online induction supported participants to develop these skills and to forge effective working relationships with academic staff. Participants appreciated the friendly, engaging and student-focussed nature of their online induction experience. To illustrate, participants explained just how important their tutors had been in helping them to overcome several pre-arrival concerns.

All the professors, the instructors, they all are wonderful. They are great. They are holding the expectation high. I feel they are all doing a good job at providing where they can for me to succeed academically. The induction set the tone for this. (P4)

I think the lecturers were very welcoming during the online induction and then also in all the teaching sessions. (P6)

The staff have been great in helping me to get to grips with academics involved in coming to university. (P8)

Several discussed how the welcoming and interactive nature of some activities helped them to start getting to know academic staff. This initial relationship building meant they felt confident enough as the semester progressed to approach them online to discuss topics relevant to their academic integration and development. This was viewed very positively by participants as evidenced in the below statements:

So, for me personally, it was a bit of a step because I came to university after a gap in my studies. About two years. So, I went travelling, and I was nervous for the academic part of it. But there were loads of supportive material online. There was insight podcasts and online induction was a great help. So, I found settling in academically fine. (P3)

I would say the academic staff are really, really nice and respectful. They have done a good job to make you feel friendly and comfortable around them when online. (P5)

The meeting I had with my personal tutor helped me feel more settled academically, because it felt like there was someone that if anything went wrong or I needed help, you could go to. And obviously they said they were going to watch grades and attendance, so it made you feel like if you needed any academic support, you could go to them. (P7)

Through getting to know staff during induction, I know I can email any of the people who teach me and they will get back to me within the day which is great and helps put you at ease. (P9)

### Theme 2: Impact of social isolation on transitioning into university

Whilst several participants referred positively to the academic focus of the programme induction period, most could not recall any specific events to promote social integration with peers and academic staff. Equally, there were some mixed experiences of social support offered by academics, especially personal tutors. For example, participant 2 reported having ‘*not really heard from them’*’ and participant 4 stating ‘*loads of people say they (personal tutors) are so good for academic and social support and things, but I have not really heard from them. And I know it is not their job to come to us, but you feel like a bit of a goose just emailing them’*’. More positively, participant 5 said ‘*there was always someone that, if anything went wrong or I needed help, you could go to’*’.

The majority believed they encountered a socially challenging transition into their first year of university. For example, participants spoke about missing out on the enriching experience that meeting, connecting and working with new people in face-to-face conditions can provide. They talked at length about lost opportunities in making new friends, discussing how they were not familiar nor especially close with many students on their programme and not feeling part of a student community. The following comments emphasise this lack of belonging and cohort identity more specifically:

Academically, I feel I have settled in well at uni, but on the social aspect, I need to improve. It is kind of strange because literally outside of my assessment presentation group, which is four guys, I have met hardly anyone else on the course, and no lecturers face-to-face. (P3)

I definitely feel like I have missed out on a lot of potential opportunities to make new friends. (P4)

Not having had much chance to make new friends in my induction and lessons at university has been tough. (P7)

It has been so hard not being able to socialise with other people on my programme and trying to get out there, make new friends, all that kind of stuff. It has been the complete opposite to that. (P8)

I have missed not being able to ask peers on things like getting to grips with certain assignments and how they are approaching a particular question. (P9)

Participants talked openly about the escalating and damaging impact of national lockdown restrictions on their general physical well-being, including poor sleep, appetite, self-esteem and confidence, as well as the wider impact on their ability to engage with academic work. The lack of in-person opportunities to socially mix within class and non-class-based settings was a cause of frustration for all. This resulted in participants feeling increasingly isolated, vulnerable, and lonely, as reflected in the following quotes:

It was hard to do anything to get to know people in person because we were not supposed to be socially integrating with anyone. I was in my flat by myself for pretty much a month. (P1)

There was no social aspect at all really, given the circumstances. I find it hard not being able to meet people, so yeah, just the lack of connection with other people. (P2)

I have not really had any social interactions with people on my course because of the whole coronavirus and lockdown situations. (P5)

The hardest thing has not been able to go on campus, and not being able to meet everyone on the course. Because I do not exactly feel like we have integrated as a group at all. (P8)

The harmful impact of social isolation is of concern across the sector, with the National Union of Students (2020) emphasising the impact of loneliness on mental health. In this study, participants specifically connected their lack of social contact and experience of online learning to their deteriorating well-being. The following comments illustrate this matter more specifically:

The lockdown has been rough to deal with, just on the social aspect really. Not so bad academically. Especially since all my flatmates went home. So, that has been kind of tough mentally. (P4)

Probably mental health. That is one main struggle I have noticed at university. I think it is probably because of not having that face-to-face interaction and literally everything is just through a screen. (P8)

It was also clear how restrictions on in-person teaching had negatively impacted on the social aspects of learning.

I quite enjoy, well, a lot of the topics that we learn about, but I feel like I would enjoy it a lot more if you were incorporating face to face practicals and you can actually speak to someone on your course in person, but you could not do that. This is the awkward thing. (P2)

When we are in an online session and we are split off into groups and you get some people who do not speak. There is the odd chance there will be someone in your group that you may know and you can talk with but some people do not even type in the chat box. (P9)

### Theme 3: Building social integration through university sports clubs and student accommodation communities

All participants discussed having felt they missed out socially on their entry into university. They especially found being unable to meet in person with peers and academic staff, both inside and outside of the classroom environment, as detrimental to their social development. However, social lives were best supported during this period by membership of strictly online university societies, sports clubs and involvement within their halls of residence communities. Participants joined these campus communities after attending formal online induction presentations delivered by university sport and student union personnel.

Despite government restrictions preventing participants from meeting up in person, it was through membership of the many online extra-curricular groups and activities which they found most helpful in building their engagement and connectedness with others. The following quotes demonstrate such findings:

I met new mates at the very start through joining a university football team and then they just happened to also be people who did my course as well. Through football was probably the best way I settled into uni, meeting a new group of people. (P1)

I joined athletics and it was fine at the start because we trained outdoors, so we had a good month of just doing that every Monday or Wednesday and we got to use the track and things like that. I think that was October time till when lockdown hit before Christmas. But that was a good part of uni. So, I am living with the girls from athletics next year. (P2)

For me, I would say joining a society and a sports team has been the best way to meet people. (P7)

I have been making new friends in my sports team who are all on different courses and are different ages and things so will be great to get to know them better when we can start mixing again, so yes, the online events for training and socials have been nice. (P8)

The role of sport and extra-curricular activities were important to the identities of participants, helping with supporting their sense of university belonging and acceptance. For many students, joining a sports club or society was one of the most memorable experiences of their time at university thus far. The following comments emphasise the nature of these findings:

Socially, it was a bit more difficult because obviously there was not the normal ways that you can meet people. But I helped make a group society for the course and found putting myself forward as course rep also helped me meet people. (P3)

I got involved in football through the information we got from induction, and it has been nice like meeting people through there even though it has not been in real life and all virtual. (P5)

I made some good networks through my university sports team, both doing the training classes over Zoom and with the social things they organise too. (P7)

For those living in university accommodation, the forming of friendships with fellow halls of residence students was much valued. Whilst not all were housed in student accommodation, those who were discussed how the friendships they had made were the highlight of their university experience so far. Often, this was with people from a range of academic programmes and with different interests and backgrounds:

Meeting my flatmates and getting to know them and everything else has been a real highpoint. We have been through so much so we will certainly always be good friends. (P1)

I feel like the most useful social activities are by far the ones done by my accommodation block. They run, like Zoom quizzes and bingos and all that stuff. So, we all do it as a flat together and then you can see other flats on there as well. (P8)

## Discussion

Universities have an important responsibility to ensure all newly arrived students encounter a successful entry into their institution. Induction activities are a central feature of the transition into university, playing an important role in ensuring students settle quickly, confidently and happily from the very beginning and feel valued, included and inspired to achieve (Turner et al. 2017). Through drawing on Student Involvement Theory (Astin 1984), this study explored the effectiveness of an exclusively online programme induction in supporting the integration of newly arrived first-year WP sports students into DRM. This inducting approach was taken as in-person programme induction was not permittable due to government restrictions imposed because of the global COVID-19 pandemic.

Within the context of Student Involvement theory, most participants felt academically but not socially involved, finding their programme induction focussed too heavily on academic rather than social engagement. This was reflected in students having a good understanding and familiarity of library facilities, resources available to support their learning and how they can access them, their ability to navigate Blackboard Ultra and a general excitement and motivation for the subject they were studying. Other popular components included staff overviewing their personal backgrounds, research specialisms, the programme and modules more generally and available mechanisms for pastoral support with personal tutors and central services.

Participants reported their transition into university had been socially very challenging, having had limited opportunities to form new friendship groups and networks in face-to-face settings and missing out on being able to orientate themselves with their campus and surrounding areas. Whilst academic integration is important for a successful transition (Allin et al. 2017), there is increasing evidence that the social aspects and extra-curricular student experiences are equally significant in establishing a sense of belonging and reducing the risk of university withdrawal, especially for those being away from home for the first time (Li and Zizzi 2018; Matheson et al. 2018). Whilst the COVID-19 pandemic has disrupted the day-to-day lifestyle and mental well-being of the general public, this has been significantly greater for many university students who had to isolate in unfamiliar household settings during periods of lock down with others they did not know well (Office for National Statistics 2020). To illustrate, the 2020 University Mental Health Survey found 20% of university students had a mental health diagnosis with those from under-represented backgrounds and facing intersectional challenges at a greater risk for lower levels of mental well-being. The study findings also highlighted the significance of extra-curricular activities in supporting the participants integration into university, their sense of belonging and ongoing mental health and well-being (Blair 2017; Preira et al. 2020). They initially learned about these clubs, events and groups through formal presentations by central services colleagues during their online induction and were strongly encouraged to join. It was through membership of online societies, sports clubs and halls of residence communities in the early stages of their university experience instead of formal online teaching sessions, that participants found most useful to socialise.

Informal peer support can often occur when students seek out and connect with fellow peers with relevant experiences (Goodman-Wilson 2021). Supporting the findings of previous studies, a reliance on friendships was found to be particularly important during the transition phase with participants who made more friends during their first term at university more likely to report better health and well-being (Klaiber et al. 2018). These study findings are consistent with features of Astin’s (1999) Theory of Involvement where fellow students and academics can prove an important resource for role modelling and enhancing the learning environment and student experience.

### Implications

The value of a programme induction process which eases the move into university and acclimatises students with their new surroundings is well supported, particularly for those who arrive from WP backgrounds (Turner et al. 2017). Several implications for designing effective post COVID-19 induction programmes emerged from the study. Firstly, we recommend reverting social elements back to being in person, so students are exposed to greater opportunities for building relationships and seeking out connections with peers on their programme, academics who teach them and the wider university community, including sports teams and societies. Secondly, as a substitute to traditional face-to-face sessions, short and informative online tutorials could be used to outline generic academic information, including overviews of module content, Blackboard Ultra and introductions from senior university management. To support greater efficiency and sustainability of practice during this time, workforce development sessions could be introduced for colleagues which provide support on developing engaging virtual resources. Thirdly, instead of placing heavy emphasis on supporting students once they arrive on campus, this could begin prior to their actual arrival during fresher’s week. We recommend that HE institutions provide prospective students with greater access to virtual campus tours, supplemented by social media information, so they can see beforehand what campuses, academic staff and facilities available are like (UCAS 2021). This would likely free up valuable time during the early and often hectic days of university for face-to-face social activities focussed on developing student belongingness, self-confidence and engagement. We also suggest that the membership benefits of campus-based communities and extra-curricular activities and the valuable role they can provide in supporting social integration continue to be widely promoted by colleagues to all students during induction programmes.

### Limitations and future research avenues

This study was not without any limitations. For example, although the recall period was short, we relied on retrospective recollections to explore participant’s experiences and views of online induction, which may be liable to lapses of memory. Interviews were conducted remotely by second-year peer mentors and participants may have decided against disclosing any negative comments with their fellow peers. Further validation of participant accounts with those from their module and personal tutors would have been advantageous. The sample was homogenous, thus limiting generalisability of findings. Data collection took place relatively early (week 10) in the participant’s university experience, hence follow-up interviews at the end of year one may have unearthed further important findings. Additional interviews could have been conducted with non-WP sports students which would have determined to what extent their experiences of online induction differed or were specific to the WP group.

## Conclusion

Undergraduate sport programmes in British universities annually recruit high numbers of WP students from diverse backgrounds. Using Astin’s Student Involvement theory as a theoretical framework, the study provides evidence on the role that online programme induction can provide in supporting first-year WP sport students to integrate into a post-92 UK university. The key study finding was that online induction was more successful in gaining academic than social engagement. Participants placed time and effort into their studies, engaged with student organisations and extra-curricular activities but had limited social interactions with other students, both from their programme and the wider university community. The main contributory factors to successful academic integration were participants sense of familiarity with academic processes and features (e.g. Blackboard ultra), their understanding of what university-level study involved and the centralised student support available (e.g. student portal).

Study findings provide colleagues working within and outside of sport disciplines with evidence to develop future online induction programme materials which best support the integrating of diverse student populations into university. These actions are likely to increase attendance to formal teaching classes, eLearning platform engagement and percentage of modules passed first time across first-year cohorts. Longer-term benefits may include improved retention, progression, satisfaction and achievement outcomes across all levels of study.

## Data Availability

The datasets used and/or analysed during the current study are available from the corresponding author on reasonable request.

## Footnotes

1 The Business and Technology Education Council (BTEC) is a vocationally based qualification which enables students to gain skills and knowledge in their subject area, then apply them in real-life and applied scenarios.

2 Blackboard Ultra is DRM's new virtual learning environment (VLE), designed to provide staff and students with a more personalised, proactive and intuitive user experience which puts teaching, learning and communication at the heart of the VLE.

3 Blackboard collaborate is a virtual classroom.
